# Connecting Health and Technology: A Comprehensive Review of Social Media and Online Communities in Healthcare

**DOI:** 10.7759/cureus.55361

**Published:** 2024-03-01

**Authors:** Pankajkumar A Anawade, Deepak Sharma, Shailesh Gahane

**Affiliations:** 1 Management, School of Allied Sciences, Datta Meghe Institute of Higher Education and Research, Wardha, IND; 2 Science and Technology, School of Allied Sciences, Datta Meghe Institute of Higher Education and Research, Wardha, IND

**Keywords:** digital health, technology integration, patient empowerment, healthcare, online communities, social media

## Abstract

This review provides an in-depth analysis of the intersection between health and technology, focusing specifically on social media's and online communities' role in healthcare. It explores the significance of these digital platforms in patient education, empowerment, and support, highlighting their potential to improve healthcare delivery and patient outcomes. Key findings are synthesized by examining existing literature, including the wide-reaching impact of social media on health information dissemination and the value of online communities in facilitating peer support. However, privacy concerns and misinformation are also addressed, emphasizing the need for careful consideration and strategic implementation of these technologies. The implications for healthcare practice and research are discussed, with recommendations for future actions and priorities outlined. Overall, this review underscores the transformative potential of social media and online communities in reshaping the healthcare landscape. It also highlights the importance of ethical and responsible use to maximize benefits.

## Introduction and background

Advancements in technology have significantly impacted the healthcare landscape, revolutionizing how patients access information, interact with healthcare providers, and manage their well-being. From wearable devices that track vital signs to telemedicine platforms enabling remote consultations, integrating technology into healthcare has improved efficiency, accessibility, and quality of care. This intersection between health and technology has opened up new possibilities for enhancing patient outcomes and transforming traditional healthcare practices [1].

Among the various manifestations of this intersection, social media and online communities have emerged as powerful tools in healthcare. Social media platforms offer a vast network for sharing health-related information, fostering peer support, and facilitating communication between patients, caregivers, and healthcare professionals. Online communities allow individuals facing similar health challenges to connect, exchange experiences, and access valuable resources. The importance of these digital platforms in healthcare cannot be overstated as they enable widespread dissemination of health information, promote patient engagement, and contribute to the democratization of medical knowledge [2].

This review aims to comprehensively explore the role of social media and online communities in healthcare. It will explore how these digital platforms are utilized within the healthcare ecosystem, examining their impact on patient education, empowerment, and support. Additionally, this review will explore the challenges and opportunities associated with integrating social media and online communities in patient care. By synthesizing existing research and offering insights into future directions, this review seeks to inform healthcare professionals, researchers, and policymakers about the potential of these technologies to enhance healthcare delivery and patient outcomes.

## Review

The role of social media in healthcare

Definition and Types of Social Media Platforms

Social media platforms are online platforms that enable individuals to create, share, and exchange information and content with others. These platforms typically host user-generated content, fostering engagement through features like likes, shares, comments, and discussions. Prominent examples of social media platforms include Facebook, Instagram, LinkedIn, Twitter, TikTok, and YouTube [3-7]. Social media platforms can be categorized into various types, including social networking, media sharing, microblogging, online forums, social bookmarking, and social news [6]. Social networking sites facilitate connections with friends and family, emphasizing person-to-person communication. Media-sharing platforms are tailored to distribute images and videos, while microblogging platforms enable sharing of concise content. Online forums serve as hubs for discussions based on shared interests or curiosity. Social bookmarking platforms allow users to save and distribute web page links, and social news platforms enable sharing of news stories and articles [4]. Overall, social media platforms provide diverse opportunities for individuals and businesses to connect, share information, and interact with others. However, they also pose challenges, such as privacy concerns and the necessity for clear guidelines and policies to ensure ethical use [3,6].

Utilization of Social Media by Healthcare Professionals

Healthcare professionals are utilizing social media platforms to engage with patients and consumers, offering them timely information, updates on outbreaks or health hazards, and avenues for providing feedback to enhance service quality [8]. Additionally, these platforms serve as channels for professional development and networking within the healthcare community, enabling professionals to share insights, debate healthcare issues, and participate in remote medical conferences [9]. Through social media, healthcare providers facilitate direct communication with patients, responding to inquiries, offering online consultations, and disseminating health advice to their followers [10].

Some healthcare organizations have integrated social media into their training processes, encouraging trainees to use specific hashtags or join groups for collaborative learning and immediate feedback on training sessions [8]. Moreover, social media plays a crucial role in marketing and public awareness efforts within the healthcare sector, promoting services, attracting new patients, and showcasing organizational achievements [8].

While the use of social media offers numerous benefits in healthcare, including improved access to health information and enhanced doctor-patient communication, it also introduces potential risks such as the dissemination of unreliable information and breaches of patient privacy rights. Hence, healthcare professionals must establish clear guidelines and policies to ensure the ethical use of social media and address privacy concerns [9,10]. By carefully managing social media usage, healthcare professionals can leverage its advantages while mitigating associated risks and challenges [8-10].

Impact of Social Media on Patient Education and Empowerment

Social media profoundly impacts patient education and empowerment in healthcare, providing patients with access to a wealth of health information, self-management programs, and online social support systems. This access contributes to improved health outcomes as patients who are better informed about their conditions are more likely to engage in self-care and adhere to treatment regimens, ultimately leading to enhanced well-being. Furthermore, social media facilitates direct communication between patients and healthcare professionals, enabling patients to seek advice and receive professional guidance online, empowering them to be more active in managing their healthcare journey [9,11,12].

Nevertheless, using social media in patient education and empowerment has its challenges. Concerns persist regarding the accuracy and reliability of health information shared on these platforms, raising questions about the credibility of online sources. Additionally, issues related to patient privacy and the potential adverse effects on doctor-patient relationships must be carefully addressed. Furthermore, considerations such as the digital divide and the importance of patients critically evaluating online information are crucial in effectively leveraging social media for patient education and empowerment [9,11,12]. Social media plays a pivotal role in patient education and empowerment in healthcare, offering benefits such as increased access to information and online support networks. However, it is imperative to tackle the challenges associated with its use, including the quality of information and patient privacy, to ensure its ethical and effective utilization in patient education and empowerment initiatives [9,11,12].

Challenges and Concerns Associated With Social Media in Healthcare

In healthcare, several challenges arise concerning the use of social media, necessitating careful consideration and strategic management by healthcare professionals and organizations. Privacy and security stand out as paramount concerns. Healthcare professionals must rigorously uphold patient confidentiality and adhere to regulations like Health Insurance Portability and Accountability Act (HIPAA). Key measures include maintaining distinct personal and professional accounts, refraining from publicly discussing patient diagnosis or treatment, and securing patient consent before sharing information [13,14]. Misinformation poses a significant risk on social media platforms, where false or misleading health information can proliferate rapidly. Healthcare entities must remain vigilant, ensuring that any information shared is accurate and verified, thus safeguarding patients from potentially harmful treatment decisions or consequences [14,15].

**Figure 1:**
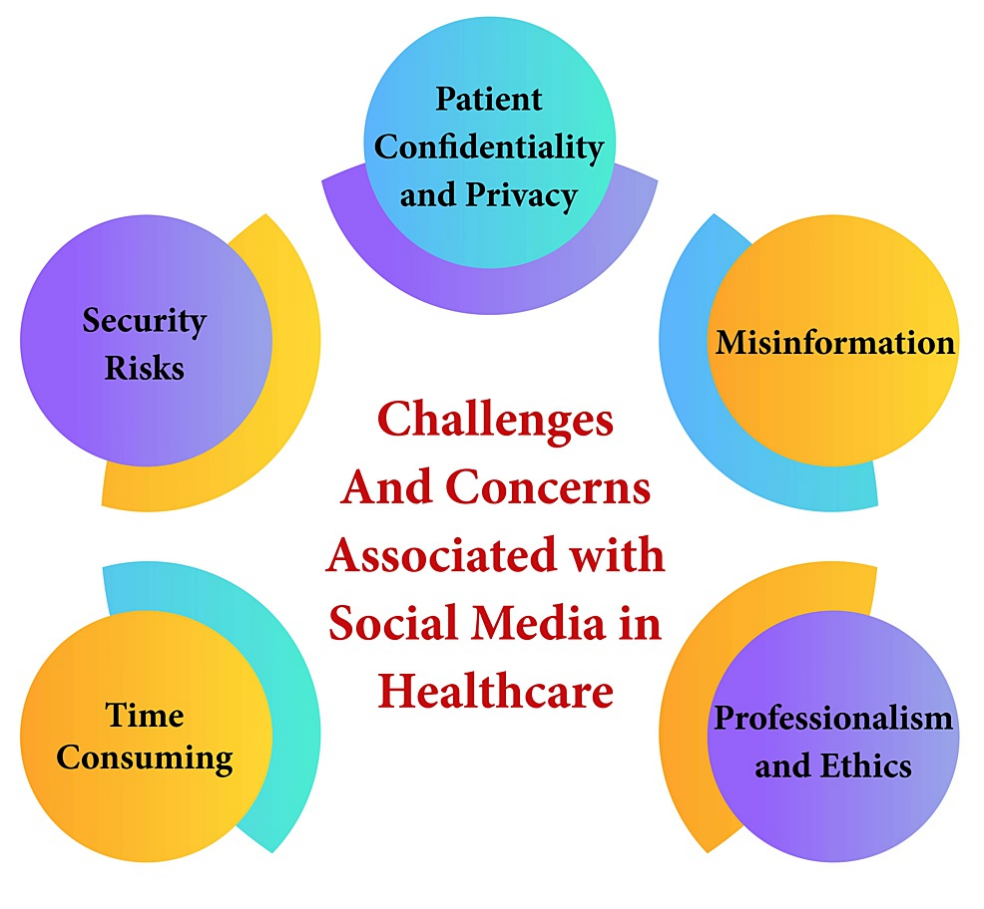
Challenges and concerns associated with social media in healthcare Image credit: This image was created by the corresponding author.

Social media can become a source of distraction and consume substantial amounts of healthcare professionals' time. This distraction may lead to errors or decreased productivity, particularly when managing professional accounts. As such, prudent time management and minimizing distractions become imperative in navigating social media's role in healthcare [13,14]. Lack of control and potential reputational damage also loom as concerns. Negative comments or reviews on social media platforms can tarnish the reputation of healthcare professionals. Thus, healthcare organizations must devise strategies to effectively manage negative feedback and maintain a positive online presence [14]. Patient confidentiality and privacy must be rigorously upheld when utilizing social media in healthcare settings. Establishing clear guidelines and providing comprehensive employee training is critical to ensure adherence to privacy rules and appropriate patient interactions [13]. To mitigate these challenges, healthcare professionals and organizations can adopt several strategies. Developing comprehensive social media policies, providing ongoing training on ethical social media use, monitoring platforms for compliance, and securing accounts to protect patient information are essential. Additionally, healthcare entities should approach social media engagement as an opportunity to foster positive patient interactions, promote accurate health information, and maintain professional reputations [13,14]. Challenges and concerns associated with social media in healthcare are shown in Figure 1.

Online communities in healthcare

Definition and Characteristics of Online Communities

An online community is a collective of individuals engaging digitally around a common interest, challenge, or objective. Typically, members of these communities share similar interests and primarily interact via the Internet. Such communities often coalesce around a particular shared interest and can span multiple online platforms. Key features of online communities include platforms for content sharing, discussion forums or newsgroups, email correspondence, chat rooms, and instant messaging. They are often termed "virtual settlements." They are characterized by four essential elements: interactivity, a diverse range of communicators, a central public space for member interaction, and sustained membership over time [16].

The hallmark characteristics of thriving online communities include their focus on fostering engagement and offering communal support, their dynamic and multifaceted nature, inclusivity, searchable content repositories, and gamification features. These communities can manifest in various forms, including private communities accessible through login or invitation only, public communities easily discoverable through search, and hybrid communities blending public and private elements, with full access requiring login credentials [17]. Virtual communities, as defined, are social groups that emerge on the internet when individuals engage in public discussions with sufficient emotional investment to establish personal relationships in the digital realm. These communities consist of people who may or may not meet face-to-face but communicate and exchange ideas through digital networks [18]. An online community gathers individuals who interact digitally around shared interests, challenges, or goals. These communities thrive on shared interests, interactivity, and sustained membership over time. Thriving online communities prioritize engagement and embrace diversity, and they can take various forms, including private, public, or hybrid configurations [17,18].

Examples of Popular Healthcare Online Communities

Patients Like Me: Patients Like Me is a prominent private and personalized health network offering patients a platform to share and access pertinent information regarding their health conditions. With over half a million active members, Patients Like Me has facilitated the publication of over 100 research studies, significantly advancing medical knowledge and patient care [19].

Health Boards: Established in 1998, Health Boards is an invaluable healthcare internet website where patients can seek guidance, support, and valuable information for further research. Covering a broad spectrum of health conditions, Health Boards has garnered popularity over the years as a trusted online community for individuals seeking health-related advice and resources [19].

Med Help: Med Help operates as a comprehensive health information-sharing portal, empowering patients to exchange quantitative data on their health, discuss symptoms, and compare treatment options. Recognized as one of the top 20 health sites by Consumer Reports Health Web-Watch, Med Help enables members to track their health journey while accessing various informative resources [19].

WebMD: Renowned as a stalwart in health information, WebMD offers many resources, including health news, medical reference content databases, interactive tools, and user communities. Widely utilized for health-related advice, support, and information, WebMD is a trusted platform for individuals seeking credible health information and resources [20].

Health Unlocked: Health Unlocked is an online patient community offering social networking services to patients and their families. Leveraging unique artificial intelligence capabilities, Health Unlocked provides customized health information and hosts over 600 health communities on its platform. By facilitating connections and providing tailored health information, Health Unlocked empowers patients and their loved ones to navigate their healthcare journey confidently [19]. These online communities provide patients invaluable platforms to seek and offer assistance, share personal experiences, and access relevant health information. By fostering connections and facilitating knowledge sharing, these communities play a pivotal role in supporting and empowering patients throughout their healthcare journeys [19].

Benefits of Online Communities for Patients and Caregivers

Online health communities offer a myriad of benefits for both patients and caregivers. These communities serve as invaluable sources of information, emotional support, and a sense of belonging. Providing access to a wealth of resources, insights, and solidarity empowers individuals and can streamline the diagnosis of common illnesses, thus reducing hospital congestion [21,22]. Through these platforms, patients and caregivers can connect with others facing similar challenges, receive crucial emotional support, and access valuable advice, ultimately leading to improved health outcomes [21,22]. Moreover, online health communities catalyze meaningful social movements and participant-driven research, enabling individuals to advocate for improved care and initiate research initiatives focused on specific medical conditions [22].

For caregivers, these communities offer invaluable access to community insights, serve as platforms for exchanging treatment ideas, and provide support from peers undergoing similar experiences [22]. Furthermore, they empower caregivers by fostering a sense of belonging and facilitating connections with like-minded individuals, especially when engagement with the local community may be challenging due to time constraints or other limitations [22]. Online health communities are indispensable in providing social support, disseminating knowledge, and nurturing a sense of empowerment for patients and caregivers [23]. With the potential to revolutionize the traditional patient-caregiver dynamic, these communities contribute significantly to enhancing the overall healthcare system [5].

Ethical Considerations in Online Healthcare Communities

Utilizing online health communities and social media platforms in healthcare brings forth a host of ethical considerations as highlighted by the Nuffield Council on Bioethics. Central to these considerations is the imperative to safeguard private information, uphold individuals' autonomy to pursue their interests, and minimize harm [24]. Moreover, integrating social media in health-related research introduces privacy, confidentiality, and consent challenges, underscoring researchers' need to meticulously address these issues and formulate precise guidelines for ethical conduct [25].

While online health communities hold promise in offering new forms of social capital for patients and healthcare services, they encounter hurdles such as privacy apprehensions, the necessity to balance the power dynamics with traditional healthcare providers, and financial and business model constraints [26]. Additionally, employing online health communities for data collection raises ethical implications, particularly regarding privacy and consent [27]. The ethical considerations encompassing online healthcare communities revolve around protecting private information, minimizing harm, and upholding confidentiality, privacy, and consent in social media for health-related research and data-gathering endeavors. Addressing these ethical concerns is imperative to unlock the potential benefits of online health communities while mitigating associated risks [24-27].

Social media and online community integration in patient care

Case Studies Illustrating the Successful Integration of Social Media and Online Communities in Patient Care

The successful integration of social media and online communities in patient care is exemplified through various case studies, showcasing the transformative potential of these platforms in healthcare delivery. One such case study is the Mayo Clinic, which has established a robust presence on social media platforms, including Facebook, YouTube, and Twitter. The clinic aims to engage diverse community stakeholders through many blog posts, podcasts, conferences, and webinars, providing an authentic voice for patients and healthcare professionals. By harnessing the revolutionary power of social media, the Mayo Clinic endeavors to build meaningful relationships and enhance patient engagement [9]. Furthermore, numerous healthcare organizations leverage social media to connect with patients, offering a spectrum of services, discounts, and newsworthy events while providing customer service and support. Studies indicate that electronic communication with patients can significantly improve care and health outcomes, facilitating enhanced access to healthcare information and educational resources [9]. Online health communities (OHCs) are invaluable platforms for patients to seek physician advice and receive professional suggestions online. Encouraging patients to utilize OHCs alongside conventional treatments can streamline diagnoses of common ailments and alleviate hospital congestion. Factors influencing patients' intentions to engage with OHCs encompass performance expectancy, effort expectancy, social influence, facilitating conditions, health awareness, and eHealth literacy [28].

Moreover, social media and online communities are pivotal in health-related research, professional development, and doctor-patient communication. These platforms offer valuable insights for research endeavors, facilitate collaboration among healthcare professionals, and support disseminating health-related information to patients. Additionally, social media serves as a conduit for delivering test results, promoting medication compliance, and soliciting patient feedback regarding healthcare services [10]. Integrating social media and online communities in patient care yields numerous benefits, including improved patient care, health outcomes, and accessibility to healthcare information and educational resources. Notable case studies such as the Mayo Clinic's innovative use of social media underscore the potential for transformative impact in healthcare delivery. Moreover, the utilization of OHCs demonstrates their efficacy in enhancing diagnostic efficiency and reducing hospital congestion. Social media and online communities support health-related research, facilitate professional development, and foster effective doctor-patient communication [9,10,28].

Tools and Strategies for Healthcare Providers to Engage With Patients Through Social Media and Online Communities

Healthcare providers have diverse social media tools to enhance networking, education, and patient care. These tools encompass social networking platforms, blogs, microblogs, wikis, media-sharing sites, and even virtual reality and gaming environments. Leveraging these platforms can facilitate professional networking, patient care, patient education, and the implementation of public health programs. For example, social media can significantly improve patient care and health outcomes by facilitating electronic communication with patients, disseminating patient education materials, and promoting health awareness initiatives. Additionally, it enables transparent communication with the community, aiding in reputation management and talent acquisition. Nevertheless, it is crucial to remain mindful of potential risks, such as the dissemination of unreliable information and infringements on patients' privacy rights. Adhering to best practices and guidelines is essential to ensure social media's practical and ethical utilization in healthcare [9,29].

OHCs serve as invaluable platforms for patients to seek advice from healthcare professionals and receive professional guidance online. The utilization of OHCs has the potential to streamline patient diagnoses and alleviate hospital congestion. Factors influencing patients' intentions to engage with OHCs encompass performance expectancy, effort expectancy, social influence, facilitating conditions, health awareness, and eHealth literacy [10]. Healthcare providers benefit significantly from harnessing social media and online communities to enhance patient care, facilitate patient education, and foster community engagement. However, it is imperative to remain cognizant of potential risks and adhere to best practices and guidelines to ensure these platforms' effective and ethical use in healthcare [9,10,29].

Patient Perspectives on the Use of Social Media and Online Communities in Their Healthcare Journey

Integrating social media into healthcare has proven beneficial for organizations, clinicians, and patients, offering many advantages. Healthcare organizations leverage social media platforms for various community engagement activities, including fundraising, customer service, news dissemination, patient education, and advertising new services. Physicians utilize online communities to access news articles and expert opinions, research medical advancements, network with colleagues, and discuss patient-related matters. On the other hand, patients benefit from social media by accessing educational resources, networking opportunities, research materials, support networks, goal-setting tools, and personal progress tracking [30].

Patients, caregivers, and healthcare professionals frequently utilize social media to form virtual communities, which serve as invaluable resources for overcoming logistical challenges, providing emotional support, and sharing disease-related information. However, it is crucial to acknowledge that the information shared on social media platforms is voluntary and may only sometimes be comprehensive or address all aspects of a research query. Patients and caregivers often use social media to learn from others' experiences, seek emotional support, and boost self-esteem. Social media narratives offer insights into patient perspectives and lifestyles, which are instrumental for understanding real-world issues in caregiving or living with a disease [31]. Despite social media's numerous benefits in healthcare, it also presents potential risks, including disseminating unreliable information and violating patients' privacy rights. Therefore, it is imperative to adhere to best practices and guidelines to ensure social media's secure and ethical utilization for healthcare information dissemination [9,31]. In conclusion, while social media and online communities play a significant role in healthcare, offering benefits such as patient education, emotional support, and access to disease-related information, it is essential to remain vigilant about potential risks and ensure the ethical use of these platforms [9,30,31].

Future directions and challenges

Emerging Trends in the Use of Social Media and Online Communities in Healthcare

The landscape of social media and online communities in healthcare is evolving rapidly, with several emerging trends shaping the industry. In 2022, healthcare professionals increasingly turn to social media platforms for various purposes, including professional development, mental health support, disease awareness campaigns, and advocacy efforts. Moreover, social media serves as a vital tool for patient education, hosting virtual conferences and events, and combating the spread of misinformation. The adoption of social audio platforms is also on the rise, providing healthcare professionals with opportunities to engage in discussions and establish themselves as thought leaders [32]. Looking ahead to 2023, several trends are poised to influence the use of social media in healthcare. These include a focus on personalization, the proliferation of video content, the integration of chatbots for enhanced patient interaction, the utilization of influencer marketing strategies, the incorporation of virtual reality experiences, the implementation of social listening tools, and the integration of augmented reality technologies. These trends aim to enhance patient engagement, deliver personalized experiences, and gain valuable insights into patient sentiment and preferences [33].

Despite the myriad opportunities social media presents in healthcare, challenges persist, such as disseminating misinformation, privacy concerns, and the imperative for stringent quality control measures. Healthcare professionals are urged to exercise discretion and utilize social media judiciously to maximize benefits while mitigating risks and enhancing patient outcomes, medical education, research endeavors, and the overall healthcare experience [34]. Furthermore, OHCs provide valuable platforms for patients to seek advice from healthcare professionals and receive professional guidance online. Encouraging patients to utilize OHCs alongside traditional treatments is essential as they can streamline the diagnostic process for common diseases and alleviate hospital congestion. Various factors influence patients' intentions to engage with OHCs, including performance expectancy, effort expectancy, social influence, facilitating conditions, health awareness, and eHealth literacy [28].

Potential Opportunities for Innovation and Collaboration

Sustainable healthcare innovation thrives through collaborative efforts and novel partnership models [35]. By fostering alliances among various stakeholders, sustainable progress can be achieved in healthcare innovation, ensuring long-term viability and effectiveness. Digital health solutions present a promising avenue for advancing health equity within healthcare systems [36]. Technology and life sciences companies can leverage digital innovations to address healthcare access and outcomes disparities, promoting more significant healthcare delivery equity.

Integrating digital technology can significantly enhance access to healthcare services [36]. By streamlining medical processes and eliminating barriers such as travel and transportation, digital solutions bridge the equity gap, making healthcare more accessible to underserved populations. Initiatives led by digital health startups aim to reduce treatment costs and enhance overall access to care. Social media is a powerful tool for fostering patient engagement within healthcare organizations [29]. By leveraging social media platforms, healthcare providers can effectively connect with diverse audiences, foster transparent communication, and raise awareness about critical public health issues. Moreover, social media serves as a platform for patient education, addressing specific patient needs, and promoting adherence to treatment regimens. Despite the benefits, social media and digital technologies pose challenges, such as misinformation and breaches of patient confidentiality [34]. Ensuring quality control through stricter regulations and ethical frameworks is imperative to mitigate these risks and safeguard patient welfare. By addressing these challenges, healthcare organizations can harness the full potential of social media and digital technologies to improve patient care and outcomes.

Addressing Privacy and Security Concerns in the Digital Healthcare Landscape

The digitization of the healthcare sector has ushered in significant cybersecurity challenges [37]. With the adoption of digital technology in healthcare, cyber threats and data security issues have become increasingly prevalent, necessitating robust cybersecurity measures to safeguard sensitive patient information. Ensuring privacy protections and transparency surrounding the use of health data is paramount for building public trust in digital medicine [38]. Transparency in health data usage and disclosures is essential to foster confidence among stakeholders and promote responsible handling of health-relevant digital data, thereby upholding patient privacy rights.

Current US health privacy laws, such as HIPAA, face limitations in adequately addressing digital technologies' privacy and security concerns [38]. There is a pressing need for comprehensive privacy and security regulations that transcend the constraints of existing laws, ensuring robust protections for health data irrespective of its origin or storage location. The concept of the digital health footprint encompasses a broad spectrum of health-related data collected from diverse sources, posing significant challenges to privacy and security [39]. Rethinking traditional definitions of health privacy and implementing appropriate safeguards is imperative to protect this expansive and varied dataset from potential breaches and unauthorized access. The increased utilization of telehealth services has exacerbated privacy and security risks within the healthcare landscape [40]. Environmental conditions, technological vulnerabilities, and operational practices contribute to the heightened exposure to cyber and technology-related data security threats in telehealth practice. Addressing privacy and security concerns in the digital healthcare realm requires a comprehensive approach [37-40]. This involves implementing stringent cybersecurity measures, enhancing privacy protections, and bridging the gaps in existing regulations. Furthermore, reevaluating the management of health-relevant data and establishing best practices for telehealth services are crucial steps toward mitigating privacy and security risks in the evolving digital healthcare landscape.

Regulatory Considerations and Policy Implications

Utilizing social media and online platforms in healthcare brings forth a range of regulatory considerations and policy implications. The rapid proliferation of digital health tools, including social media and mobile health (mHealth) apps, has underscored the need for more stringent safeguards, enhanced measures for enforcing compliance, and rigorous and transparent regulation to bolster public trust. In the United States, initiatives beyond the purview of the FDA are emerging, exemplified by the Federal Trade Commission's (FTC's) policy statement mandating that digital health apps adhere to specific regulations concerning user notification of privacy breaches and obtaining explicit consent for data usage [41]. Regarding healthcare professionals' utilization of social media, advantages and risks exist. While social media is an effective platform for professionals to communicate and advocate for public health, it also presents risks such as disseminating unreliable information, breaches of patients' privacy rights, and potential legal ramifications. Therefore, formulating guidelines delineating appropriate usage by healthcare providers is imperative, with healthcare organizations tasked with establishing employee policies to mitigate the risks associated with social media usage [9].

From a regulatory standpoint, using social media and mHealth for collecting and transmitting patient-generated health data is becoming increasingly prevalent. A comprehensive understanding of the legal and regulatory considerations surrounding data ownership and usage empowers clinicians to align their practices and make well-informed decisions regarding adopting data-sharing technologies. Ensuring transparency concerning data ownership and usage policies is pivotal, enabling patients and providers to make informed choices regarding creating and transmitting patient-generated health data [42]. Integrating social media and digital health tools into healthcare necessitates clear regulatory standards, transparency, and robust safeguards to safeguard patient privacy, data security, and compliance with regulations. Healthcare organizations and professionals must establish unequivocal guidelines and policies to address the risks of using social media and online platforms effectively [9,41,42].

## Conclusions

In conclusion, this review has comprehensively examined the role of social media and online communities in healthcare. Through analysis, it became evident that these digital platforms play a crucial role in patient education, empowerment, and support. Social media platforms are powerful tools for disseminating health information globally, while online communities offer a valuable space for individuals to connect with others facing similar health challenges. However, challenges such as privacy concerns and misinformation underscore the importance of careful consideration and strategic implementation of these technologies. The implications for healthcare practice and research are substantial as professionals can leverage these platforms to enhance patient engagement, improve health outcomes, and extend the reach of their services. Moreover, researchers can explore innovative ways to harness these technologies for health promotion and patient-centered care. Moving forward, it is essential to prioritize research initiatives to understand the impact of social media and online communities on patient outcomes and healthcare delivery. Efforts should also address privacy, security, and misinformation challenges, collaborating with healthcare organizations and policymakers to develop guidelines that promote ethical and responsible use of these platforms. Additionally, investments in digital literacy and education programs can empower patients and healthcare professionals to navigate the digital landscape safely and effectively. By taking these actions and prioritizing research in this area, we can harness the transformative potential of technology to enhance healthcare delivery and improve patient experiences.
